# Improved Access to Behavioral Health Care for Patients in a Large New York City Behavioral Health Clinic by the Transition to Telemedicine

**DOI:** 10.1089/tmr.2024.0060

**Published:** 2025-03-31

**Authors:** Aaron Reliford, Emily Zhang, Anni Liu, Olga Lanina, Sharifa Z. Williams, Navin Sanichar, Shabana Khan, Isaac Dapkins, William Gordon Frankle

**Affiliations:** ^1^ Department of Child and Adolescent Psychiatry, NYU Grossman School of Medicine, New York, New York, USA.; ^2^ Department of Psychiatry, NYU Grossman School of Medicine, New York, New York, USA.; ^3^ Division of Social Solutions and Services Research, Center for Research on Cultural and Structural Equity in Behavioral Health, Nathan S. Kline Institute for Psychiatric Research, Orangeburg, New York, USA.; ^4^ Department of Population Health, NYU Grossman School of Medicine, New York, New York, USA.

**Keywords:** telemental-health, telemedicine, behavioral health access, federally qualified health center, visit completion rate

## Abstract

**Objective:**

To examine the transition to telemental health within the behavioral health program of a large federally qualified health center, The Family Health Centers at NYU Langone, in the 3 months following the onset of the COVID-19 pandemic—specifically impacts on show rates and access to care.

**Methods:**

Demographic and clinical information for all scheduled visits was collected for two time periods: the telemental health period, March 16, 2020-July 16, 2020 (46,878 visits, 5,183 patients), and a comparison period, March 15, 2019-July 16, 2019 (47,335 visits, 5,190 patients). Data collected included modality, appointments scheduled/completed/cancelled/no-showed, age, gender, race, language, and diagnosis. Generalized estimating equations with a compound symmetry correlation structure and logit link were used for analysis.

**Results:**

An ∼twofold increase in the likelihood of completing a visit in 2020 vs. 2019 (adjusted OR = 1.92, *p* < 0.001) was observed. Patients who received treatment in both time frames (*n* = 2,961) also showed increased completion rates in 2020 vs. 2019. No diagnostic group had a decline in competition rate from 2019 to 2020, including those with severe mental illnesses, although patients with schizophrenia were significantly less likely to complete an initial visit in 2020 compared with 2019 (adjusted odds ratio, aOR = 0.37, *p* < 0.001). For those with appointments in both timeframes, we noted a significant association between gender and completion rate in 2019 (male 66.5% ± 25.1% vs. female 64.2% ± 24.4%, ANOVA *p* = 0.01), which was eliminated by implementation of telemental health.

**Conclusions:**

This study supports the use telemental health to increase access for all patients, including those from under-represented, lower socioeconomic status backgrounds.

## Introduction

The COVID-19 pandemic served as an impetus for legal and regulatory flexibilities at the federal and state level, allowing for the rapid expansion of telehealth, including telemental health—the use of telemedicine to provide mental health assessment and treatment at a distance through audio or audio-visual means.^1–3,6^ While the pandemic significantly accelerated the use of telemental health encompassing telepsychiatry and teletherapy, this modality has been in use for over 60 years^4^ with support for use across a variety of populations, disorders, and settings.^5–9^ Importantly, research indicates concordance in diagnosis, risk assessment, and intervention recommendations between telemental health and in-person care across settings, including emergency departments (EDs) and primary care clinics.^7,8,10,11^

Appointment completion rates are challenges in medicine that can hinder the progress of treatment. Commonly reported reasons for missing appointments include scheduling conflicts, caregiving responsibilities, transportation issues, and lack of motivation.^12^ Notably, during the COVID-19 pandemic, 41% of U.S. adults reported having missed or delayed in-person appointments due to pandemic-related concerns, such as fear of contracting the virus or closed facilities.^13^ Telemental health can overcome these barriers to treatment by allowing patients to save money/time on traveling to the clinic, which reduces conflicts with work or caretaker roles.^14^

Improved appointment completion rates with telehealth have been highlighted in reports from various groups. For instance, a study of adult patients in a large academic outpatient psychiatry clinic in North Carolina demonstrated a significant decrease in the no-show rate for telemental health visits during the first 10 months of the COVID-19 pandemic (March to December 2020) compared to the no-show rate for in-person visits during the 10 months before March 2020.^12^ Similar findings of reductions in no-show rates using telemental health during the COVID-19 pandemic have been noted in primary and specialty care clinics in Ohio^15^ and in academic clinics in Massachusetts.^16^

The behavioral health service of the Family Health Centers at NYU Langone, a Federally Qualified Health Center (FQHC), is located in the Sunset Park area of Brooklyn and serves approximately 13,000 patients with over 125,000 visits per year. Sunset Park has a population of ∼132,000, largely lower socioeconomic status, 48% born outside the United States, 49% with limited English proficiency, and 22% without health insurance.^17^ The population served within behavioral health reflects the community: those from under-represented, lower socioeconomic status backgrounds—an important factor to highlight given barriers to access for telemental health in such populations.^16^ Prior to the COVID-19 pandemic, almost all visits were in person, and the use of telemental health within the behavioral health service line was limited to two pilot projects, one for patients in homeless shelters and one to bring child psychiatry to public schools. While multiple factors accounted for the limited use of telemental health in this setting, key among these included the regulatory steps necessary to complete the telemental health approval through the New York State Office of Mental Health and the inability for an FQHC to bill their Prospective Payment System (PPS) rate in situations where the patient was at home.^18^

The onset of the COVID-19 pandemic prompted the Family Health Centers at NYU Langone’s behavioral health services to rapidly transition the majority of clinical services to telemental health within a two-week period in March 2020. Simultaneously, the federal Public Health Emergency resulted in a dramatic change in the regulatory process for implementing telemental health in NYS, and subsequent modification of guidance included the capacity for FQHCs to bill their PPS rate when both the providers and the patients were at home.^19^ This study explores the impact of this rapid service transition during the initial phase from March 2020 to July 2020 and examines accessibility and engagement as compared to traditional in-person care pre-Covid in 2019 during the same months, as directly measured by visit completion rates compared between these 2 time periods. We aim to evaluate whether telemental health can effectively maintain and possibly enhance access to mental health care and to identify potential disparities that may need to be addressed to ensure equitable access for all patients.

## Methods and Materials

After approval by the NYU Grossman School of Medicine IRB, a review of the electronic medical record (EMR) was performed to collect demographic and clinical information on all behavioral health visits over two times. The first was the telemental health implementation period between March 16, 2020 and July 16, 2020, and the second was the comparison dataset prior to this implementation, pre-COVID-19, between March 16, 2019 and July 16, 2019. The data collected included the modality (telemental health vs. in-person care) and the number of appointments that were scheduled (initial or follow-up visits), completed, canceled, and no-shows. Patients’ age (>/= 5 years old), self-identified gender, race, preferred language, as well as the primary psychiatric diagnosis associated with each appointment, were also extracted. The primary diagnoses were categorized into the major DSM-5 diagnostic categories.^20^

### Statistical analysis

Independent variables of interest included sociodemographic characteristics and diagnostic information, such as patient age, gender, race, preferred language for care, and primary psychiatric diagnosis.

Data for all behavioral health clinic visits were used to compare visits completed, canceled, and no-showed between 2019 and 2020 using generalized estimating equations (GEE) with a compound symmetry correlation structure and logit link. Three models were fit, one for each outcome—visit complete (0/1), visit canceled (0/1), and visit no-show (0/1)—with year of visit as the primary predictor and adjusting for month, gender, race, preferred language, and primary psychiatric diagnosis. Comparisons in completion rates, the percentage of scheduled appointments that were successfully completed (i.e., number of completed visits/number of scheduled visits), between 2019 and 2020 for those patients who had visits during both years were performed with a paired *t test* or one-way ANOVA.

A *p* < 0.05 level was used for determining statistical significance. Analyses were conducted using SAS version 9.4 (Cary, NC) and IBM SPSS Statistics version 28 (Armonk, NY).

## Results

### Comparing 2020 versus 2019

During the study period (March 16 to July 16) in 2020, there were a total of 46,878 patient visits scheduled for 5,183 unique patients. During the same time period in 2019, there were 47,335 patient visits scheduled for 5,190 unique patients (Table 1). About two-thirds of the clinic visits in 2020 (62.2%) were telemental health visits (either audio/video or audio alone), representing 4,304 unique patients; there were no telemental health visits in 2019. Table 1 presents the completion, cancellation, and no-show rates in both years as well as results from GEE analysis for all patients and, separately, patients under and over 18 years old. After adjustment for sociodemographic characteristics and primary diagnosis, model results found a significant, nearly twofold higher odds of completing any visit in 2020 compared to 2019 (adjusted odds ratio, aOR = 1.92, *p* < 0.001). The odds of a canceled visit (aOR = 0.41, *p* < 0.001) and no-show visit (aOR = 0.78, *p* < 0.001) were lower in 2020 vs. 2019. Both adult and pediatric patients demonstrated a significantly higher odds of completing visits in 2020 vs. 2019; however, the aOR was 2.13 for adults vs. 1.41 for children.

**Table 1.** Behavioral Health Visits for All Patients in 2020 and 2019, *n* (%)

Visit characteristics	Year		aOR	^a^ *p*
	2020	2019		
Total visits scheduled	46,878 (100.0)	47,335 (100.0)	—	—
Number of unique patients	5,183 (100.0)	5,190 (100.0)	—	—
Number of unique patients (<18 yo)	1,348 (26.0)	1,234 (23.8)	—	—
Number of unique patients (>18 yo)	3,835 (74.0)	3,956 (76.2)	—	—
Visits cancelled	4,053 (8.7)	9,426 (19.9)	0.41	<0.001*
Visits no-show	7,050 (15.0)	9,227 (19.5)	0.78	<0.001*
Visits completed—All Patients	35,771 (76.3)	28,653 (60.5)	1.92	<0.001*
Visits completed—Patients <18 yo	10,819 (74.8)	7,466 (65.1)	1.41	<0.001*
Visits completed—Patients ≥18 yo	24,952 (77.0)	21,187 (59.1)	2.13	<0.001*
Telemental health visits	29,157 (62.2)	0 (0.0)	—	—
Number of unique patients	4,304 (100.0)	—	—	—

^a^ *p* is the significance value from GEE model adjusting for visit month, gender, race, preferred language, and primary diagnosis between 2020 and 2019.

No difference was noted in the proportion of females to males who had completed visits in 2020 when compared with 2019 (*X*^2^ = 3.08, df = 1, *p* = 0.08). We did note a significant difference in patient race comparing 2020 to 2019, with a greater proportion of individuals identifying as White, African American/Black, and Pacific Islander in 2020 and a lower proportion of individuals identifying as Asian, Other, or Unstated categories in 2020 (*X*^2^ = 16.2, df = 6, *p* = 0.01). A notable finding when comparing the completion rates for racial groups was that, after adjustment, the odds of a completed visit were significantly decreased among adults identified as African American/Black (aOR = 0.90, *p* = 0.018), although significantly increased among children identified as African American/Black in 2020 (aOR = 1.21, *p* = 0.009).

In order to explore differences in patients who were continuing their care (i.e., existing patients) during the transition to telemental health vs. those who initiated care during this transition (i.e., new patients), we examined two areas. First, we compared the completion rates for new visits vs. follow-up visits (Table 2) for both years for all patients. The odds of completing initial (aOR = 1.39) and follow-up visits (aOR = 1.93) were significantly higher in 2020 compared to 2019. Higher completion rates for both types of visits were seen in both adults and pediatric patients. However, for pediatric patients, the higher completion rate was not statistically significant for initial visits (aOR = 1.19, *p* = 0.329). The only finding related to diagnosis was that the odds of completing an initial visit were significantly decreased (aOR = 0.37, *p* < 0.001) among adults with a primary diagnosis of schizophrenia, whereas the odds of completing follow-up visits in this group were significantly increased (aOR = 1.24, *p* < 0.001).

**Table 2.** Comparison of the Completion Rates for Initial and Follow-Up Visits

Visit characteristics	Year		aOR	^a^ *p*
	2020	2019		
Initial visits	65.4%	52.6%	1.39	<0.001*
Patients <18 yo	62.8%	53.0%	1.19	0.329
Patients ≥18 yo	66.4%	52.5%	1.43	<0.001*
Follow-up visits	76.7%	60.8%	1.93	<0.001*
Patients <18 yo	75.2%	65.4%	1.42	<0.001*
Patients ≥18 yo	77.3%	59.3%	2.13	<0.001*

^a^ *p* is the significance value from GEE model adjusting for visit month, gender, race, preferred language, and primary diagnosis between 2020 and 2019.

We also analyzed the data for patients who received care during both time frames to assess for potential selection bias in those who participated in telemental health. A total of 2,961 patients had appointments in both 2020 and 2019 during the target timeframe (March 16 to July 16), allowing for within-patient comparison. Table 3 lists the overall completion rate for these 2,961 patients as well as the completion rate by gender, race, preferred language, and diagnosis. The average completion rate was 76.4% ± 24.8% and 65.0% ± 24.7% in 2020 and 2019, respectively (paired *t-test*, *p* < 0.0001). In both years, a significant relationship between race and completion rate was observed (2019: F = 4.6, df = 6, *p* < 0.001; 2020: F = 4.2, df = 6, *p* < 0.001). All groups showed statistically significant higher completion rates with the implementation of telemental health. Asian patients had statistically significantly higher completion rates in 2020 when compared with African American, Other, Pacific Islander, and White patients, and in 2019 when compared with Other, Pacific Islander, and White patients. In both years, a significant relationship between preferred language and completion rate was observed (2019: F = 5.7, df = 6, *p* < 0.001; 2020: F = 3.4, df = 6, *p* = 0.003), as well as between diagnosis and completion rate (2019: F = 2.1, df = 9, *p* < 0.03; 2020: F = 3.3, df = 9, *p* < 0.001). With regards to diagnosis, those with trauma-related disorders, substance use disorders, and impulsivity disorders did not exhibit a significantly higher rate of completion. On the other hand, those with serious mental illness, including depressive disorders, bipolar illness, and schizophrenia, all demonstrated a significantly higher visit completion rate when telemental health was utilized. Finally, a significant association between gender and completion rate was observed in 2019 (male 66.5% ± 25.1% vs. female 64.2% ± 24.4%, one-way ANOVA F = 6.0, df = 1, *p* = 0.01), which was not observed in 2020 (male 75.9% ± 25.6% vs. female 76.6% ± 24.4%, one-way ANOVA F = 0.6, df = 1, *p* = 0.44), suggesting that the gap in completion rates between men and women was eliminated with the implementation of telemental health.

**Table 3.** Completion Rates for Patients Seen in Both Years (*n* = 2961)

	2020	2019	^a^p
Overall completion rate	76.4% ± 24.8%	65.0% ± 24.7%	<0.0001
Completion rate by gender			
M (*n* = 1063, 35.9%)	75.9% ± 25.6%	66.5% ± 25.1%	<0.0001
*F* (*n* = 1897, 64.1%)	76.6% ± 24.4%	64.2% ± 24.4%	<0.0001
Completion rate by race			
Asian (*n* = 150, 5.1%)	83.9% ± 22.1%	73.2% ± 18.9%	<0.0001
Unknown (*n* = 294, 9.9%)	77.1% ± 24.8%	66.1% ± 25.9%	<0.0001
White (*n* = 1391, 47.0%)	77.1% ± 24.3%	65.0% ± 24.9%	<0.0001
Native American (*n* = 50, 1.7%)	75.8% ± 26.0%	62.1% ± 25.7%	0.003
African American/Black (*n* = 285, 9.6%)	75.6% ± 25.0%	66.6% ± 25.5%	<0.0001
Pacific Islander (*n* = 169, 5.7%)	74.3% ± 24.3%	63.7% ± 24.0%	<0.0001
Other (*n* = 622, 21.0%)	73.5% ± 26.3%	62.2% ± 24.1%	<0.0001
Completion rate by preferred language			
Chinese—Mandarin (*n* = 44, 1.5%)	84.2% ± 24.0%	74.7% ± 15.5%	0.03
Chinese—Cantonese (*n* = 39, 1.3%)	84.2% ± 21.8%	83.7% ± 14.9%	0.89
English (*n* = 1885, 63.7%)	76.3% ± 24.2%	64.1% ± 24.9%	0.0001
Spanish (*n* = 966, 32.6%)	75.7% ± 26.2%	65.7% ± 24.4%	0.0001
Arabic (*n* = 9, 0.3%)	65.2% ± 32.9%	56.9% ± 35.7%	0.45
Unstated (*n* = 3, 0.3%)	63.3% ± 12.0%	65.8% ± 08.0%	0.85
Other (*n* = 14, 0.5%)	94.9% ± 12.0%	63.8% ± 25.5%	0.0005
Completion rate by diagnoses			
Schizophrenia/psychotic disorders (*n* = 271, 9.2%)	79.7% ± 24.0%	69.6% ± 23.4%	<0.0001
Neurodevelopmental disorders (*n* = 233, 7.9%)	78.1% ± 22.8%	62.8% ± 27.2%	<0.0001
Bipolar and related disorders (*n* = 231, 7.8%)	79.0% ± 23.5%	62.4% ± 24.5%	<0.0001
Anxiety disorders (*n* = 494, 16.7%)	77.2% ± 24.3%	65.8% ± 24.5%	<0.0001
Mood disorder, NOS (*n* = 86, 2.9%)	76.6% ± 26.7%	61.2% ± 24.1%	<0.0001
Depressive disorders (*n* = 1355, 45.8%)	75.9% ± 25.0%	64.8% ± 23.7%	<0.0001
Trauma-related disorders (*n* = 203, 6.9%)	70.9% ± 26.6%	66.4% ± 27.7%	0.07
Substance related diagnoses (*n* = 11, 0.4%)	72.4% ± 25.3%	58.8% ± 30.0%	0.22
Disruptive/impulsive disorders (*n* = 34, 1.1%)	63.1% ± 23.7%	61.3% ± 31.4%	0.79
Other (*n* = 43, 1.5%)	74.2% ± 30.0%	63.5% ± 25.2%	0.08

^a^ *p* is the significant value from paired *t test*.

## Discussion

In this study, we examined the rapid transition to telemental health within the behavioral health program of a large federally qualified health center, The Family Health Centers at NYU Langone, in the ∼3 months following the onset of the COVID-19 pandemic in New York State. One of the concerns raised during the transition to telemental health was whether this modality would provide the ability to serve our patient population to the same degree as in-person care. The current study shows that access to care was maintained and even enhanced in some cases with the near-complete transition to telemental health during the study period from March 2020 to July 2020.

### Comparing 2020 versus 2019

We observed a ∼twofold increase in the likelihood of completing a visit in 2020 when compared with the same time frame in 2019, during which all appointments were carried out in person. This was the result of a significant reduction in the likelihood of cancellations and no-shows (Table 1). These findings are consistent with previous reports showing similar decreases in cancellation rates and increases in visit completion rates when telemental health was compared with face-to-face care.^21,22^ Attendance was also improved, reflecting results in the more recent literature, which has demonstrated greater ease of access with telemental health, particularly when providers and patients were restricted to being at home during the pandemic.^21,22^ Telehealth reduces costs and time associated with travel and decreases chances of exposure to the COVID-19 virus.

### Racial differences

We did note a significant difference in patient race comparing 2020 to 2019, with a greater proportion of individuals identifying as White, African American/Black, and Pacific Islander in 2020 and a lower proportion of individuals identifying as Asian, Other, or Unstated categories in 2020. A notable finding when comparing the completion rates for racial groups was that, after adjustment, the odds of a completed visit were significantly decreased among adults identified as African American/Black, although significantly increased among children identified as African American/Black in 2020.

### New versus follow-up visits

One aspect we were particularly interested in examining was whether access to care was impacted by this transition, which we explored in two ways. First, we looked at completion rates for initial visits (i.e., new treatment) vs. follow-up visits (i.e., those already established in care) for both adults and children. It was important to examine differences between existing patients and new patients to explore potential challenges in adapting to the telehealth platform. Both groups were not familiar with the telehealth platform, and analyzing the completion rates can highlight potential areas for improvement. Existing patients who have an established relationship with clinicians may have high trust and engagement in care; therefore, analyzing the completion rates for the transition of care to telehealth can offer clear insights into technological challenges and telehealth operational efficiency. Our results show increases in the completion rates of both initial and follow-up visits across both groups. This may indicate technical challenges or unfamiliarity with the telehealth platform was not a barrier for new and existing patients. However, the increase for initial visits in children overall was lower (aOR = 1.19) and not statistically significant, perhaps suggesting unique challenges in using telemental health with children. We also noted that patients with a diagnosis of schizophrenia were significantly less likely to complete an initial visit in 2020 when compared with 2019 (aOR = 0.37, *p* < 0.001), but had increased follow-up completion rates in 2020 similar to those of other clinic patients. This result is consistent with previous findings that the population with serious mental illness faced challenges to access care even before the pandemic, such as low income, poor insurance access, unstable housing, low education level, and cognitive impairment.^23^ However, the increased follow-up completion rates in 2020 suggest that, once engaged in care, patients with schizophrenia were equally able to access and utilize telehealth resources.

### Patients with appointments in both years

The second route we took to explore the engagement in care was examining if patients who were already in treatment with our program were able to maintain their care during this transition. We looked at the subset of patients who had scheduled appointments during the study timeframe in both 2020 and 2019. As shown in Table 3, this subset of patients displayed increases in completion rates in 2020 when compared with 2019. However, patients with primary diagnoses of trauma-related disorders, substance-related disorders, or impulsivity disorders did not show a statistically significant increase in completion rate in 2020 vs. 2019. Of note, the analysis may have been impacted by the low number of patients in these diagnostic categories. It is important to note that for patients already in care, none of the diagnosis groups had a decline in the completion rates from 2019 to 2020, including patients diagnosed with severe mental illnesses such as schizophrenia and bipolar illness.

### Gender differences in completion rates

Finally, for those with appointments in both years, we noted a significant association between gender and visit completion rates in 2019, with males having higher visit completion rates. The implementation of telemental health eliminated this association, such that in 2020, males and females had nearly identical completion rates (75.9% vs. 76.6%, respectively). Interestingly, female patients had significantly higher satisfaction scores for telemental health than males. These data suggest that access to care for female patients is disproportionately impacted when care is provided solely with an in-person modality. It is likely, in our patient population, that females, who, in addition to working, often serve as the main caregivers for children and/or elderly parents, have more barriers to attending in-person visits. Supporting this are reports that reduced need to travel and ease of rescheduling are the main drivers of patient satisfaction with telemental health and adherence to appointments.^16,24,25^

## Conclusion

Taken together, these data support the hypothesis that telemental health increases access to mental health care for patients, including those from under-represented, lower socioeconomic status backgrounds, with some caveats. First, it was surprising to see that African American/Black adults completed appointments at lower rates in 2020 than in 2019, while African American/Black children had significantly increased visit completion rates in 2020. Mirroring these findings, in a recent study comparing appointment completion rates at a group of academic clinics between May and June of 2019 vs. 2020 after implementation of telehealth, Franciosi et al.^16^ found a decreased proportion of non-white patients and reductions in non-English-speaking patients seen via telehealth, suggesting potential differences in telehealth access. More work needs to be undertaken to understand these findings and ensure that care can be accessed by all. Less surprising was the finding that patients with schizophrenia showed a significant decline in completion rates for initial intake visits, as individuals with psychotic disorders often have difficulty in engaging in care for a variety of reasons. However, it is encouraging that ongoing care does not appear to have been disrupted by this transition, as the completion rates for follow-up visits for those with schizophrenia were significantly increased, similar to the increase seen for other diagnostic categories.

This study has several limitations. First, we do not have data to compare in-person care with telemental health on clinical outcomes. While many published studies support the use of telemental health as providing equivalent outcomes to in-person care,^6–11^ some studies do not. The purpose of the current study was not to directly compare clinical outcomes but rather to determine whether access to care was maintained with the transition to telemental health. We did explore ED visits as a proxy for clinical outcomes; however, as the study period in 2020 corresponded to the early months of the pandemic, ED visits for non-COVID-related issues were low across the board, which was reflected in both our population and in other studies.^26^ Additionally, it might have been even more informative to include a third comparison time point to determine whether there are ongoing benefits of telemental health use or stratification of the visit completion subgroups by visit type (i.e., initial vs. follow-up) as it relates to diagnosis or other demographics. This we hope to explore in future research. Another limitation is the self-selecting nature of the study participants, as patients who did not have routine access to the technology needed for telemental health or could not utilize the technology may have made up a disproportionate number of the visit no-shows or cancellations. Future studies will be needed to better understand the specific technological limitations for patients to ensure all patients have equal access to care. Finally, we would want in future studies to fully stratify whether other factors could be considered (i.e., complexity of patients) as reasons for differences in show rates and access to care.

## Abbreviations Used

EDs: emergency departments
EMR: electronic medical record
FQHC: Federally Qualified Health Center
GEE: generalized estimating equations
